# Response to critique by Finch and Murray (2025): Misshot kangaroos and the limits of welfare regulation

**DOI:** 10.1017/awf.2025.10045

**Published:** 2025-11-03

**Authors:** Daniel Ramp, Dror Ben-Ami

**Affiliations:** 1 Centre for Compassionate Conservation, Transdisciplinary School, University of Technology Sydney, Australia; 2 Compassionate Conservation Middle East, The Steinhardt Museum for Natural History, Tel Aviv University, Israel

**Keywords:** Commercial harvest, Kangaroos, Shooting accuracy, Compliance, Welfare standards

We thank Finch and Murray (2025) for engaging with our earlier article exploring the welfare costs to kangaroos killed during commercial harvesting operations (Ben-Ami *et al.* 2014). In their commentary, Finch and Murray question the validity of evidence cited in relation to shooting accuracy by commercial harvesters. We applaud any scrutiny of compliance by shooters in meeting the National Code of Practice (AgriFutures Australia 2020), which requires “harvesters must aim to shoot target kangaroos and wallabies in the head (so as to destroy the vital areas of the brain)”. While some issues associated with the claims made by Finch and Murray (2025) have already been addressed by the editors of *Animal Welfare* (Nielsen & Rodenburg 2025), we write to address the merit of specific criticisms being made of Ben-Ami *et al.* (2014). In doing so, we use this critique to highlight broader concerns that the welfare implications of the commercial killing of kangaroos cannot be adequately evaluated or assured in conditions where adherence to regulation is unenforceable at the point of kill.

## Veracity of studies examining misshot kangaroos

The authors contrast two non-peer-reviewed surveys: one conducted by the RSPCA (2002) on behalf of Environment Australia, and another by Ben-Ami (2009) on behalf of Animal Liberation NSW. Finch and Murray (2025) are concerned about how the data from Ben-Ami (2009) were represented in Ben-Ami *et al.* (2014). They claim that we wrote that those carcases that “were not cut through the atlantal-occipital joint … [are] then assumed to mean the animal was not shot in the head”, and that we then state “that 40% of harvested kangaroos are not shot in the head.” This is a clear misreading of our work — we stated that “that up to 40% of kangaroos per chiller may have been neck shot”— acknowledging that we are unable to categorically determine adherence to the Code of Practice because of the additional removal of the neck as well as the head. It remains plausible that up to 40% of inspected carcases may have been misshot as noted by Ben-Ami (2009).

Further, they claim that a major difference between Ben-Ami (2009) and RSPCA (2002) is that the latter was not only credible, but validated. This dismissal of the former in favour of the latter appears without evidence — our reading of the RSPCA (2002) report identified no instance of validation. Instead, Finch and Murray (2025) rely on *ad hominem* remarks to discredit findings from those they do not wish to be given standing. This rhetorical strategy constitutes a fallacy of vice — discrediting evidence on the grounds of alleged bias rather than by assessing methodology. By this reasoning, pro-industry research would also be invalidated. Sound scientific evaluation must rest on methods, not ideology.

## Methodological assumptions and limitations

Both the RSPCA (2002) and Ben-Ami (2009) studies provide partial and imperfect insights into shooting accuracy. The RSPCA report explicitly acknowledged the difficulty of detecting bullet wounds on carcases with skins intact but without heads, the limitations of small-scale direct observation, and the inability to account for kangaroos left in the field. Critically, findings of these types of surveys should be used with caution for two primary reasons.

Firstly, they represent only those bodies with observed wounds, for which we can be certain that death did not adhere to the Code of Practice and whose welfare was compromised. Unlike the qualifier “may have been neck shot” we added to Ben-Ami *et al.* (2014), the RSPCA (2002) report states that all inspected carcases without observed wounds were classed as ‘head-shots’, but the assumption that these individuals died according to the Code of Practice does not hold. Carcases and skins with unobserved wounds could either have (i) been misshot but wounds were missed (Type 2 error, false negative), (ii) received head shots which did not lead to instantaneous death (i.e. non-brain head/neck shots), or else (iii) they could have received head shots leading to instantaneous death (i.e. brain shots). As it stands, it is impossible to know what the true distribution of these plausible scenarios was for the carcases and skins not assigned to the body-shot category.

Moreover, carcase-based surveys exclude individuals discarded due to being misshot, kangaroos that escaped wounded, pouch young killed as collateral, and the broader family units affected (as discussed in Ben-Ami *et al.* 2014). These systematic omissions underestimate welfare impacts. Industry practices, such as decapitation in the field, further compromise detection of jaw, muzzle, and neck wounds. The Ben-Ami (2009) survey sought to address some of these gaps, though no study to date has provided a reliable population-level estimate of compliance with the Code of Practice.

## Implications for welfare regulation

The central issue is not which dataset should be privileged, but rather that the commercial kangaroo industry currently operates without enforceable oversight at the point of kill. The Code of Practice requires instantaneous death from a brain shot, yet neither the RSPCA nor Ben-Ami studies demonstrate this is consistently achieved. All we know is that at least 4% are misshot, but evidence suggests that the true number could be much higher. Until robust, independent monitoring mechanisms are developed, it cannot be credibly claimed that welfare standards are being upheld.

It is incumbent upon regulators and industry to provide transparent and verifiable evidence of compliance. Without such proof, continued reliance on commercial kangaroo shooting as an acceptable practice is ethically indefensible. We thank the authors for engaging with our work and encourage further scrutiny of shooting accuracy and welfare outcomes, where progress can only be made through rigorous methodological analysis.
